# Unsoundness of Mind Not Always Insanity

**Published:** 1907-06-08

**Authors:** G. H. Savage

**Affiliations:** Ex-President of the Neurological Society, Past President of the Medico-Psychological Association, Consulting Physician to Guy's Hospital, and late Lecturer on Mental Diseases


### Hospital Clinics.

### "UNSOUNDNESS OF MIND NOT ALWAYS INSANITY."

### Notes of a Lecture,

By G. H. SAVAGE, M.D.Lond., M.B.,F.R.C.P.Lond., Ex-President of the Neurological Society, Past President of the Medico-Psychological Association, Consulting Physician to Guy's Hospital, and late Lecturer on Mental Diseases.

A good deal of mental unsoundness exists that has no right to be considered insanity. The Commissioners in Lunacy have maintained, and still do maintain even to the bitter legal end, that lunacy and unsoundness of mind are equivalent terms; and you, as general practitioners, in signing a certificate, have to say that the person under consideration is a person of unsound mind—that is, a lunatic. From this arise three considerations. First, from the medical point of view, what do we mean by insanity? Unsoundness of mind is the dictionary equivalent, but from the legal point of view this is an error; for in law it is a question as to whether a man understands the nature and quality of his acts. The legal question is one of responsibility. Society, on the other hand, inquires whether the person is dangerous to himself or others; if so, then he ought to be shut up. Some of the popular papers, and even papers of good repute, consider that a man may be in a very bad mental state, but unless he is dangerous to himself or to others he should not be locked up.

Twelve months ago a man was shut up in an asylum on the usual lunacy certificates. He demanded that his insanity should be inquired into by a jury. The trial cost thousands of pounds, and in the result the jury found that he was a person of unsound mind, and he was so adjudged. Those of the public Press immediately took up the matter. They said, "Here is a man of unsound mind, who has certain vague ideas as to clothing, ventilation, and other eccentric notions about his singing and his vocal powers. Why should he—an otherwise harmless creature—be shut up simply on the dictum of one or two specialists?" The newspapers maintained that such a man, though of unsound mind, need not be locked up.

There is unsoundness of mind that need not be treated as insanity; there are many epileptics who "have bees in their bonnets" who need not nevertheless be sent to an asylum, and it is to such people I wish to refer to-day. I have said over and over again that no absolute rule can be laid down, for insanity is purely a relative term. There is no micro-organism of insanity. There is no insane bacillus, nothing specific that is going to make a definite change in the nervous system, or produce a definite alteration of conduct. The brain has only got a certain number of ways of expressing its maladies. Brain decay, or brain poisoning, or simple inharmonious working—each may produce similar symptoms in some cases.

Whether the symptoms are to be treated as insanity or not depends, to quote another axiom of mine, upon the length of the purse. If a wealthy man is eccentric it does not matter. Should he prefer to have his breakfast at midnight he can have a night staff instead of a day staff to look after him. But the same conduct in another man who ought to be earning his living, or who has no servants to look after him, will have a different practical significance. Though the mental condition is the same, the cost of private care in the latter instance is out of relationship to the man's means, and society therefore interferes.

It is possible you may be called to see people who are either mad or delirious. A favourite axiom of mine is, never certify an alcoholic as "lunatic"; that is, avoid certifying a man suffering from delirium tremens. The only medico-legal regrets I have reason to feel in regard to my profession have been in connection with "alcoholics." To the alcoholic "all things are possible," and however bad the mental symptoms may be, the whole may clear off, and apparent recovery occur. A person does not fully recover until he recognises his malady, and the persistent alcoholic never does this.

Then there are many instances of people who suffer from forms of delirium associated with specific fevers such as typhoid—people who have malarial troubles and who have taken a certain amount of liquor and got run down and passed into a chronic delirious condition. In such cases be cautious in deciding whether the patient is delirious or mad. Some persons who have suffered from repeated attacks of delirium may pass into a state of chronic hallucinations in which they seem to lead a double life. So that if left alone they are busy talking to the "invisibles," while at the same time they may be recalled to the work-a-day world. Some such are mad enough, but do not necessarily need seclusion. Some mental disorders depend directly on the condition of other functions. Thus I saw lately a young fellow who had suffered from malaria in Central Africa. When I saw him he had the wildest and most general exaltation of ideas, and was wishing to start the most extravagant business. This was associated with a pulse of 120 without rise of temperature. Rest in bed and suitable tonics reduced his pulse to normal and his mind to sanity, but a too early return to work caused a relapse with similar pulse-rate and with the old extravagance. In the end rest cured him. This form of mental disorder was entirely dependent upon the physical conditions. Delusions with exaltations may be also associated with diabetes and phthisis. In such cases the mental symptoms may mask the physical condition entirely. I have had occasion, in consultation in a case of melancholia and refusal to eat, to ask a medical man in attendance to examine the urine. It was loaded with sugar, showing that the melancholia and the refusal to eat were due to diabetes that had not been recognised. Such mistakes are injurious to the practitioner, as persons resent having to take their relatives to "mad doctors" when they are only suffering from physical disease. Kidney disease is also a factor in slight mental disorders. These are all practical points, and one has to ask how many of these cases are "insanity." The unsoundness of mind is not due primarily to brain disease in these cases, and what the practitioner has to do is to consider and treat the bodily disease rather than the mental condition.

There is such a thing as defect of brain that cannot be called insanity. Every week I see weak-minded giants sixteen or seventeen years of age, six feet, or even more, in height. They can not be treated as lunatics, and yet there is the defective mind. They are defective in control, and very often indolent and totally unable to apply themselves to work. They have no delusions and no hallucinations, and yet they are useless logs, and give rise to a great deal of trouble.

I saw yesterday a young fellow who had been rather brilliant at one of the universities. Although a steady fellow living in college and not unhealthy-minded, occasionally playing football and golf, he was one who absolutely could not learn. He was well brought up and well educated. He said to me, "I *cannot* learn"; nor can he at present. There is no insanity about the lad, though his memory is gone. There is an abnormal condition of the brain, but it is not insanity. I have known many brilliant fellows who fail like this, who develop sleeplessness and begin with morphine, which soon finishes them. You recognise this in adolescents who outgrow their strength; they are incapable of control and require rest. Any attempt at restraint gives rise to the most violent outbreak of rage, during which all sorts of things may be destroyed. This is a peculiar nervous disorder, but may not amount to insanity, and is called hysterical. Yet if the youth takes a "header" out of the window the coroner's jury will be sure to ascribe the occurrence to temporary insanity, and you will be blamed. Some forms of vicious self-indulgence may indicate mental disorder, which is not necessarily insanity. Regarding the occasions on which these disorders result in offences against society I used to be ready and willing, as part of my professional duty, to defend people who were weak in mind in this direction; but I came to the conclusion that, as Maudsley said of them, there was some madness and some badness, but often more badness than madness. If they were more bad than mad I did not like to have anything to do with them. Sometimes when elderly men get punished for these offences in many cases the condition will not be accepted as insanity by lawyers, as the man knows what he is doing.

Speaking of moral faults, kleptomania is a common one. Children begin by thinking that their parents belong to them, and not they to their parents. They think that the whole world belongs to them. Some children never can be taught that everything they can touch, reach, and move does not necessarily belong to them. There are people who never get beyond this infantile stage. Certain others—women at the climacteric period, for example—are most frequently given to this stealing, and in a very great number of cases they steal perfectly useless things; yet they know they are doing wrong, and you have the greatest difficulty in persuading juries that such unsound persons ought not to be punished. Social crimes of the sexual type and the indecent type are examples of cases on the borderline of mental disorder without actual insanity.

Then there is another type known as social misfits; and some of these are equally difficult to deal with, because there is so little to go upon. I was asked by a lady whether I would mind calling to see her husband. She complained that he neglected her, was not sober, and treated her badly in many ways. He had been a good husband for a quarter of a century, and she was quite sure that he was going out of his mind. Some weeks afterwards a gentleman came to me, saying that he was very anxious about his wife. He said she had lost her affection, was not sober, and had completely changed in her ways. I told him that I had already seen her, and that she was asking the same questions about himself. I have no doubt that the one who consulted me first was the nearer to insanity. The last trial that Lord St. Helier had before him was one in which a specialist had been consulted by both husband and wife about their respective insanities. In very many of these cases the weakness is associated with the climacteric change—a change so complete, bodily and mentally, that she sees everything wrong and gets into a state of uncertainty and "wobble."

Again, there may be over-growth of a certain tendency. An educated man, coming from a very neurotic stock, suddenly loses his self-control, and sends libellous postcards to his relations and to others. He can control himself sufficiently at times, but periodically he is not responsible, yet not insane. In such a case society steps in and says that if such acts are not physically dangerous they are morally dangerous, and therefore such a person should be shut up, and I am not disinclined to act along that line in some cases. A great deal of mental disorder that cannot be treated as insanity is hysterical—a loss of control, almost resembling mania. I recall one case admitted to Bethlem Hospital—fanciful, hysterical, and paraplegic. When the paraplegia disappeared she became slightly maniacal. She left the hospital still hysterical, still paraplegic, though there was improvement in both conditions. In such conditions one meets with false accusations. These conditions are improved by an interval of isolation, after which the patient will admit that there is not a word of truth whatever in the accusations. A man was admitted into Bethlem Hospital in consequence of threats to murder his wife, whom he accused of adultery. He appeared to recover, but said the accusation was well founded and true, but he would separate from her and live with his married son. Later he came to tell me that the whole was a delusion, and that his wife was as honest as possible. Yet there was a time when he appeared sane in conduct, but had a fixed false idea. There may be the greatest difficulty in determining what is sanity and what is insanity; in fact, what is the truth. It is not a very unusual thing to hear of such accusations being made against the doctor or the husband, and many have been brought before me. I was once asked to see a young lady who was at one of the universities. One Sunday morning when the students were going to chapel she gave them the slip and walked to London. Her parents received a telegram from the authorities: "Your daughter has disappeared We do not know what has become of her." When she arrived home she seemed confused, and alleged that when she was a mile or two out of town she received the inspiration that she was to be the virgin-mother of the second Christ, and she was met by a tramp, to whom she told the tale. He said, "Well, then, I will be the father." She was put to bed and carefully nursed, and in a few months she got quite well, there being no truth in the tramp story. Here was a case of mental unsoundness undoubtedly, and yet there would be great difficulty in treating it as insanity.

Cases of self-injury also belong to this group. A girl consulted a dermatologist about a peculiar worm which she said was appearing on her skin. She had a peculiar mark on different parts of her body, but always within the reach of her right hand. She had been applying liquor potassæ.

One of the disorders which have sometimes to be treated as insanity is loss of recent memory associated with symptoms of alcoholism. Of the two most typical cases I have had to deal with, one was an English peer who had a good many attacks of delirium tremens, and at last passed into a condition of complete loss of recent memory, so that from moment to moment he did not remember what he had said or what was passing, and yet he was agreeable company to spend a day or two with. When I asked who is the Prime Minister or even Queen he would not know. Here was a man absolutely defective in recent memory, and yet with perfect memory of who and what he was. When it was suggested that he should be served with a notice that the Court of Chancery would conduct his affairs I declined to allow him to be considered a lunatic. On the other hand, a woman suffering similarly, belonging to the lower middle class, had to be sent to the asylum because she had no one to look after her and protect her.

Conditions of mental defect which scarcely amount to insanity constantly arise. They may depend upon alcohol, illness, or senility. With senility you may get dementia so advanced that the man is totally unfit to manage himself or his affairs, or you may get simple placid dementia in which the man is not capable of managing his affairs, but can look after himself fairly well. Senile troubles may show as (1) indecent exposure, obscenity and things of that kind. In such cases the French law regards senility as mitigating responsibility. (2) But you may get hallucinations with senility, and in such cases I admit it is extremely difficult to know when to treat cases of this kind as insane, and when to treat them as not requiring certification or detention. Thus a man may have marked hallucinations of sight with signs of senility, but otherwise he may be as clear mentally as possible. This may lead to very serious trouble. If such a man imagined he saw burglars in his house and fired out of his windows and killed someone, should he be tried as a criminal or as a lunatic?

There may be disorders of the nervous system which interfere seriously with the social well-being, and yet they can hardly be treated as insanity. (3) With age and physical weakness come also sometimes suspicion, inquisitiveness, and mental uncertainty. The persons cannot make up their minds to do anything, not even to leave a room without asking whether there is another door. That need not be treated as insanity in the well-to-do, but it is different if a man has to earn his living.

One recognises a great deal of mental disturbance which may lead to insanity, but should not always or necessarily be treated as insanity and certified. It is pretty certain that we are very near the time when there will be a considerable relaxation in lunacy legislation in this direction. It will be a more reasonable legislation. At present the lunacy certificate says that unsound mind and lunacy are equivalent. They are not equivalent, and I want you to recognise that.

